# Homeostasis model assessment of insulin resistance in relation to the poor functional outcomes in nondiabetic patients with ischemic stroke

**DOI:** 10.1042/BSR20180330

**Published:** 2018-05-08

**Authors:** Siou Li, Changhao Yin, Weina Zhao, Haifu Zhu, Dan Xu, Qing Xu, Yang Jiao, Xue Wang, Hong Qiao

**Affiliations:** 1 Department of Endocrinology, The Second Affiliated Hospital of Harbin Medical University, Harbin 150086, China; 2 Department of Neurology, Hongqi Hospital of Mudanjiang Medical University, Mudanjiang 157011, China; 3 Department of Endocrinology, Hongi Hospital of Mudanjiang Medical University, Mudanjiang 157011, China; 4 Heilongjiang Provincial Key Laboratory of Cerebral Ischemic Stroke Prevention and Control. Mudanjiang 157011, China

**Keywords:** functional outcome, insulin resistance, ischemic stroke, prognosis

## Abstract

Whether insulin resistance (IR) predicts worse functional outcome in ischemic stroke is still a matter of debate. The aim of the present study is to determine the association between IR and risk of poor outcome in 173 Chinese nondiabetic patients with acute ischemic stroke. This is a prospective, population-based cohort study. Insulin sensitivity, expressed by the homeostasis model assessment (HOMA) of insulin sensitivity (HOMA index = (fasting insulin × fasting glucose)/22.5). IR was defined by HOMA-IR index in the top quartile (Q4). Functional impairment was evaluated at discharge using the modified Rankin scale (mRS). The median (interquartile range) HOMA-IR was 2.14 (1.17–2.83), and Q4 was at least 2.83. There was a significantly positive correlation between HOMA-IR and National Institutes of Health Stroke Scale (*r* = 0.408; *P*<0.001). In multivariate analyses, patients in IR group were associated with a higher risk of poor functional outcome (odds ratio (OR) = 3.23; 95% confidence interval (CI) = 1.75–5.08; *P*=0.001). In multivariate models comparing the third and fourth quartiles against the first quartile of the HOMA-IR, levels of HOMA-IR were associated with poor outcome, and the adjusted risk of poor outcome increased by 207% (OR = 3.05 (95% CI 1.70–4.89), *P*=0.006) and 429% (5.29 (3.05–9.80), *P*<0.001). In a receiver operating characteristic curve (ROC) analysis of poor outcome, the area under the curve (AUC) increased from 0.80 to 0.84 (95% CI: 0.79–0.88) by adding HOMA-IR to clinical examination variables (*P*=0.02). High HOMA-IR index is associated with a poor functional outcome in nondiabetic patients with acute ischemic stroke.

## Introduction

In China, the annual stroke mortality rate is approximately 1.6 million, which has become the leading cause of deaths and adult disability [1]. The age-standardized incidence and mortality rates were 1114.8 per 100000 people, 246.8 and 114.8 per 100000 person-years, respectively [2]. Early and accurate prediction of outcome in stroke is important and influences risk-optimized therapeutic strategies. Thus, it is important to identify those stroke patients at high risk for poor outcome.

Insulin resistance (IR), the proposed main pathophysiological mediator of metabolic syndrome [3], may lead to a prothrombotic and proinflammatory state, characterized by a derangement in endogenous fibrinolysis and increased platelet activation [4]. IR is a precursor and mechanism of type 2 diabetes and is associated with the development of atherosclerosis and hypercoagulability [5]. IR is a pivotal pathophysiologic contributor to the increased risk of cardiovascular disease (CVD) [6–9]. In contrast, no statistically significant association was observed in the Rotterdam Study (using the homeostasis model assessment (HOMA)-IR model) [10].

Previous studies had suggested that measures of IR have been associated with stroke risk in nondiabetic adults [5,11,12], but whether IR-related metabolic disturbances may have an impact on the prognosis of acute ischemic stroke once it has occurred remains largely unknown. Interestingly, a previous study reported that IR predicted incident post-stroke depression in Chinese subjects from ischemic stroke [13]. We hypothesized that IR, reflected by HOMA-IR model, would be associated with higher risk of worse outcomes amongst nondiabetic, stroke adults. We designed a prospective study to test this hypothesis in 173 Chinese nondiabetic patients with acute ischemic stroke.

## Materials and methods

From February to December 2016, consecutive nondiabetic subjects with ischemic stroke admitted to the Department of Emergency of the Second Affiliated Hospital of Harbin Medical University, China, were identified. All patients were admitted within 24 h of experiencing a new focal or global neurological event. The study population was exclusively Chinese. Brain Computed Tomography (CT) and/or MRI were performed in all the patients. Specific additional inclusion criteria for the present study comprised: (i) availability of blood samples to determine fasting insulin and glucose 24 h after admission, (ii) no chronic treatment with insulin before qualifying stroke, (iii) no diabetes before admission (diabetes at baseline was defined as use of insulin or oral hypoglycemic drugs, fasting serum glucose >7.0 mmol/l, random serum glucose <11.1 mmol/l or 2-h serum glucose <11.1 mmol/l), and (iv) admission glycemia of <7.0 mol/l. In addition, patients with malignant tumor, head trauma, liver and kidney dysfunction, severe edema, autoimmune diseases, and without informed consent were also excluded. The present study has been approved by the ethics committee of the Second Affiliated Hospital of Harbin Medical University. All participants or their relatives were informed of the study protocol and their written informed consents were obtained.

Clinical information was collected. Demographic data (age and sex), body mass index (BMI), and history of risk factors (hypertension, hyperlipidemia, CVD, smoking habit, and alcohol abuse) were obtained at admission. Pre-stroke therapy (oral anticoagulants, and statins) and acute treatment (intravenous thrombolysis and/or mechanical thrombectomy) were recorded. Clinical severity was assessed at admission using the National Institutes of Health Stroke Scale (NIHSS). Functional impairment was evaluated at discharge using the modified Rankin scale (mRS). A good functional outcome of stroke patients was defined as an mRS score of 0–2 points, while poor functional outcome was in the range of 3–6 points [14]. Strokes were classified according to the criteria of the TOAST (Trial of Org 10172 in Acute Stroke Treatment) classification [15]. The clinical stroke syndrome was determined applying the criteria of the Oxfordshire Community Stroke Project (OCSP) [16].

For all the patients, blood samples were collected on the first day of admission under fasting state and within 48 h of symptom onset (within 0–6 (*n*=38), 6–12 (*n*=42), 12–24 (*n*=39), and 24–48 (*n*=54) h from symptom onset). Serum was immediately separated by centrifugation at 3500 rpm for 15 min. Right away, the insulin level was measured with a commercial ELISA (ARCHITECT insulin assay; Abbott, Wiesbaden, Germany) and other biochemical parameters (triglyceride, low and high-density lipoprotein, fasting blood glucose (FBG) and high-sensitivity C-reactive protein (Hs-CRP) were assessed using ROCHE COBASC311 (ROCHE, Basel, Switzerland). Determinations were performed in duplicate, and the mean value of both determinations was used. The mean intra-assay coefficients of variation were less than 10% in all cases. IR was quantitated with the HOMA-IR following the formula described by Matthews et al. [17]: HOMA-IR = fasting serum insulin (mU/l) × FBG (mmol/l)/22.5.

### Statistical analysis

The results were expressed as percentages for categorical variables and as medians (interquartile ranges (IQRs)) for continuous variables. The Mann–Whitney U test and chi-square test were used to compare the two groups. Spearman’s Rank correlation was used for bivariate correlations. In addition, associations between HOMA-IR and NIHSS score were also assessed using ordered logistic regression models in multivariate adjustment with possible confounders.

The influence of HOMA-IR on poor functional outcome was performed by multivariate binary logistic regression analysis, which allows adjustment for those significant risk factors in univariate logistic regression analysis. Results were expressed as adjusted odds ratios (ORs) with the corresponding 95% confidence interval (CI). For a more detailed exploration of the HOMA-IR and poor functional outcome, we also used multivariate analysis models to estimate adjusted OR and 95% CIs of poor functional outcome for HOMA-IR quartiles (with lowest HOMA-IR quartile as reference). In addition, the relationship between patients in IR group (HOMA-IR Q4 compared with Q1-3) and poor functional outcome was also calculated.

Further, receiver operating characteristic curves (ROC) were used to test the overall accuracy of the HOMA-IR and other markers to predict functional outcome. Integrated discrimination improvement (IDI) and net reclassification improvement (NRI) indices were calculated to determine the clinical utility of the addition of HOMA-IR to established risk factors and the ability of HOMA-IR to improve unfavorable outcome or mortality prediction [18]. All statistical analysis was performed with SPSS for Windows, version 20.0 (SPSS Inc., Chicago, IL, U.S.A.). Statistical significance was defined as *P*<0.05.

## Results

In the present study, we recorded 173 nondiabetic subjects with stroke. Overall median age was 65 (IQR: 58–70) and 53.2% were male in the study population. The median time from stroke onset to inclusion in the study was 5.0 (IQR: 2.0–9.0) h. Similarly, the median time from stroke onset to blood collected was 13.5 (IQR: 8.0–25.5) h. The median NIHSS score was 11 (IQR: 7–16). The median (IQR) HOMA-IR index was 2.14 (1.17–2.83). A value of 2.83 was the cut-off point of the HOMA-IR index Q4 distribution (the IR group). A HOMA-IR value greater than 3 was present in 21.4% (37/173) of participants. In the present study, 23 patients (13.3%) received thrombolysis treatment. Interestingly, HOMA-IR in patients who received thrombolysis treatment were not significantly different compared with those who did not receive thrombolysis treatment (2.32 (IQR: 1.53–2.77) compared with 2.13 (1.14–2.84); *P*=0.53).

Vascular risk factor characteristics stratified by IR (HOMA-IR Q4 compared with Q1–Q3) are presented in Table 1. Individuals in IR group (HOMA-IR Q4) were younger; more likely had higher blood pressure, BMI, NIHSS score, triglyceride levels, FBG, and Hs-CRP levels; and had lower HDL cholesterol levels. They were less likely to be physically active, and more likely suffered from hypertension, hypercholesterolemia, and a history of transient ischemic attack (TIA). In addition, HOMA-IR were with no differences between sexes, stroke subtype distribution, stroke etiology, and pre-stroke treatment (*P*>0.05, respectively). Interestingly, there was a significantly positive correlation between HOMA-IR and NHISS (*r* = 0.408; *P*<0.001). Further, there was still a significant positive trend between HOMA-IR and NIHSS score (*P*=0.003), using ordered logistic regression after multivariate adjustment for possible confounders: age, sex, BMI, infarct volume, time from onset to blood collection, stroke syndrome, stroke etiology, pre-stroke treatment, physical activity, vascular risk factors, and serum levels of Hs-CRP, FBG, HDL, LDL, and triglycerides.

**Table 1:** Vascular risk factor profile of the study population

Variable	HOMA-IR Q1–Q3 (*n*=130)	HOMA-IR Q4 (*n*=43)	*P*^1^
Age at baseline, median (IQR)	66 (59–70)	62 (56–68)	<0.05
Male, *n* (%)	69 (53.1)	23 (53.5)	NS
BMI, median (IQR), kg/m^2^	26.8 (24.9–28.3)	29.6 (28.1–31.1)	<0.01
NIHSS score	10 (7–15)	16 (11–20)	<0.001
Time from stroke onset to inclusion, h, median (IQR)	4.5 (2.0–8.0)	6.0 (2.5–10.5)	<0.05
Time from stroke onset to blood collected, h, median (IQR)	12.5 (9.5–17.5)	15.0 (11.0–25.0)	<0.05
Prior vascular risk factors, number (%)			
Hypertension	67 (51.5)	33 (76.7)	<0.01
Hypercholesterolemia	43 (33.1)	20 (46.5)	<0.05
CVD	28 (21.5)	10 (23.3)	NS
Previous TIA	20 (15.4)	13 (30.2)	<0.01
PVD	11 (8.5)	4 (9.3)	NS
Smoking	26 (20.0)	9 (20.9)	NS
Blood pressure, median (IQRs), mmHg			
Systolic	140 (132–148)	145 (135–155)	<0.05
Diastolic	81 (77-87)	86 (81–93)	<0.01
Moderate-heavy physical activity, *n* (%)	36 (27.7)	5 (11.6)	<0.01
Pre-stroke treatment, number (%)			
Antiplatelet agents	28 (21.5)	9 (20.5)	NS
Statins	17 (13.1)	6 (14.0)	NS
Laboratory findings, median (IQR)			
FBG, mmol/l	5.15 (4.89–5.48)	5.77 (5.38–6.03)	<0.001
Hs-CRP, mg/dl	0.50 (0.32–0.64)	0.56 (0.38–0.77)	<0.01
Triglycerides, mmol/l	1.56 (1.32–1.79)	1.89 (1.56–2.05)	<0.01
Cholesterol level, mmol/l			
HDL	1.10 (0.95–1.25)	0.94 (0.83–1.19)	<0.01
LDL	2.75 (2.55–3.07)	2.80 (2.59–3.12)	NS

Abbreviations: HDL, high-density lipoprotein; LDL, low-density lipoprotein; NS, No Significance.

1 *P*-value was assessed using Mann–Whitney U test or Chi-square test.

**Figure 1:**
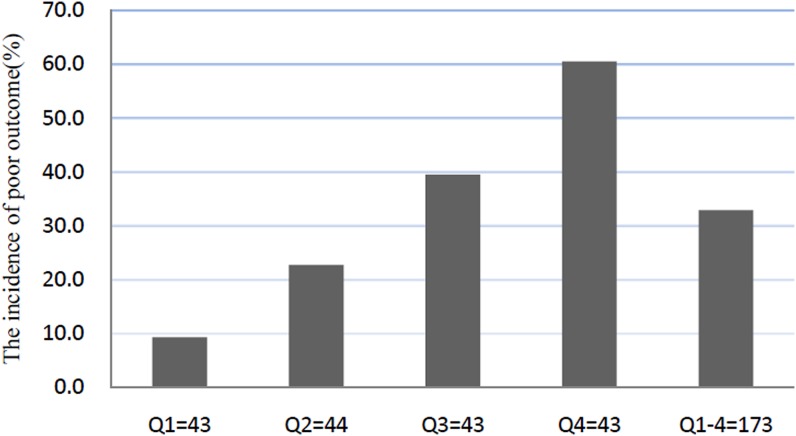
The incidence for poor functional outcomes according to the baseline HOMA-IR quartiles HOMA-IR in Quartile 1 (<1.17), Quartile 2 (1.17–2.14), Quartile 3 (2.15–2.83), and Quartile 4 (>2.83). Poor functional outcome was defined as mRS as 3–6.

**Figure 2:**
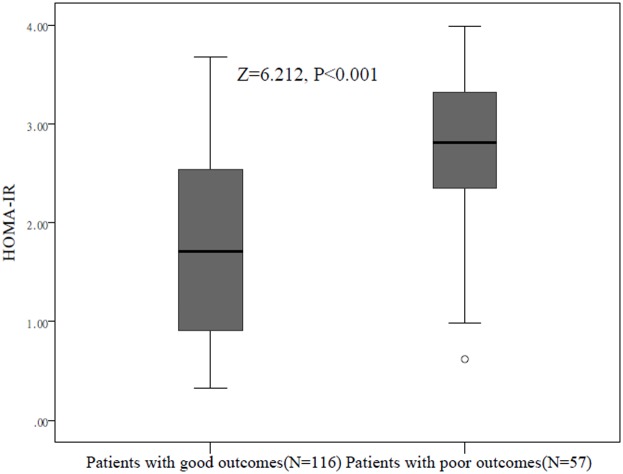
Distribution of HOMA-IR in stroke patients with poor functional outcomes and good functional outcomes Horizontal lines represent medians and IQRs. *P*-values refer to Mann–Whitney *U* tests for differences between groups. Poor functional outcome was defined as mRS as 3–6.

At discharge, a poor functional outcome was found in 57 patients (32.9%; 95% CI: 25.9–40.0%) with a median mRS score of 4 (IQR: 3–6). The poor outcome distribution across the HOMA-IR quartiles ranged between 9.3% (first quartile) and 60.5% (fourth quartile), Figure 1. HOMA-IR in patients with a good outcome were significantly lower than those in patients with a poor outcome (1.71 (IQR: 0.90–2.55) compared with 2.81 (IQR: 2.25–3.34);* Z* = 6.212; *P*<0.0001; Figure 2). In univariate logistic regression analysis, we calculated the ORs of HOMA-IR as compared with the NIHSS score and other risk factors. With an unadjusted OR of 3.43 (95% CI: 2.21–5.33), HOMA-IR had a strong association with poor functional outcome. After adjusting for all other significant outcome predictors in univariate logistic regression analysis (Table 2), HOMA-IR remained an independent poor outcome predictor with an adjusted OR of 2.45 (95% CI: 1.59–4.07). In multivariate analyses, patients in IR group (HOMA-IR Q4) were associated with a higher risk of poor functional outcome (OR = 3.23; 95% CI = 1.75–5.08; *P*=0.001). After adjusting for other established risk factors in univariate analysis, in multivariate models comparing the third and fourth quartiles against the first quartile of the HOMA-IR, levels of HOMA-IR were associated with poor outcome, and the adjusted risk of poor outcome increased by 207% (OR = 3.05 (95% CI: 1.70–4.89), *P*=0.006) and 429% (5.29 (3.05–9.80), *P*<0.001), respectively (Table 3). The independent association of HOMA-IR with poor outcome was confirmed using the likelihood ratio test (*P*=0.002). In addition, age, BMI, infarct volume, the NIHSS score, and blood levels of FBG and Hs-CRP remained significant outcome predictors, unlike all others assessed (Table 2).

**Table 2:** Univariate and multivariate logistic regression analysis for poor outcome

Parameter	Univariate analysis		Multivariate analysis^1^	
	OR (95% CI)	*P*	OR (95% CI)	*P*
Age (increase per unit)	1.15 (1.06–1.27)	<0.001	1.10 (1.04–1.18)	0.003
Male sex	1.28 (1.03–1.58)	0.26	—	
BMI (increase per unit)	1.18 (1.05–1.30)	0.002	1.12 (1.04–1.25)	0.02
Infarct volume (increase per unit)	1.20 (1.08–1.33)	0.001	1.16 (1.04–1.27)	0.01
NIHSS score (increase per unit)	1.45 (1.21–1.66)	<0.001	1.37 (1.18–1.53)	0.002
Time from onset to blood collection (increase per unit)	0.97(0.90–1.05)	0.13	—	
Stroke syndrome				
TACS	1.66 (0.65–3.22)	0.22	—	
PACS	1.15 (0.81–1.83)	0.75	—	
LACS	0.81 (0.47–1.66)	0.51	—	
POCS	0.59 (0.32–1.40)	0.39	—	
Stroke etiology				
Small-vessel occlusive	0.64 (0.38–1.33)	0.45	—	
Large-vessel occlusive	0.94 (0.68–1.27)	0.33	—	
Cardioembolic	1.15 (0.94–1.46)	0.09	—	
Other	1.22 (0.98–1.77)	0.26	—	
Pre-stroke treatment (Yes compared with No)	0.93 (0.88–0.99)	0.03	0.95 (0.87–1.05)	0.09
Acute-stroke treatment (Yes compared with No)	0.85 (0.79–0.92)	0.006	0.91 (0.85–0.95)	0.01
Moderate-heavy physical activity	1.87 (1.13–2.56)	0.02	1.64 (1.05–2.68)	0.06
Blood pressure (increase per unit)	0.96 (0.90–1.13	0.12	—	
Vascular risk factors (Yes compared with No)	1.44 (1.02–2.01)	0.04	1.36 (0.88–2.15)	0.18
Hs-CRP (increase per unit)	1.14 (1.03–1.22)	0.008	1.11 (1.04–1.20)	0.01
FBG (increase per unit)	1.06 (1.02–1.14)	0.005	1.03 (1.01–1.07)	0.008
HDL (increase per unit)	0.66 (0.50–1.05)	0.10		
Triglycerides (increase per unit)	1.18 (0.92–1.77)	0.15		
HOMA-IR	3.43 (2.21–5.33)	<0.001	2.45 (1.59–4.07)	<0.001

Abbreviations: HDL, high-density lipoprotein; LACS, lacunar syndrome; PACS, partial anterior circulation syndrome; POCS, posterior circulation syndrome; TACS, total anterior circulation syndrome.

1 Includes the significant risk factors in univariate logistic regression analysis for multivariate logistic analysis (age, BMI, infarct volume, NIHSS score, pre-stroke and acute treatment, physical activity, vascular risk factors and serum levels of Hs-CRP and FBG).

**Table 3:** Logistic regression model for HOMA-IR using poor functional outcomes as the dependent variables, after adjustment by possible risk factors^1^

Dependent variables	OR (95% CI)^2^	*P*-value
Poor functional outcomes^3^		
HOMA-IR (1° quartile)	Reference	─
HOMA-IR (2° quartile)	1.85 (1.07–3.12)	0.061
HOMA-IR (3° quartile)	3.07 (1.70–4.89)	0.006
HOMA-IR (4° quartile)	5.29 (3.05–9.80)	<0.001

Abbreviations: HDL, high-density lipoprotein; LDL, low-density lipoprotein.

1 Includes the significant risk factors in univariate logistic regression analysis for multivariate logistic analysis (age, BMI, infarct volume, NIHSS score, pre-stroke, and acute treatment, physical activity, vascular risk factors and serum levels of Hs-CRP and FBG). Poor functional outcome was defined as mRS in 3–6.

2 The likelihood ratio test (*P*=0.002).

3 HOMA-IR in Quartile 1 (<1.17), Quartile 2 (1.17–2.14), Quartile 3 (2.15–2.83), and Quartile 4 (>2.83).

Based on the ROC, the optimal cut-off value of HOMA-IR as an indicator for auxiliary diagnosis of poor outcomes was projected to be 2.53, which yielded a sensitivity of 73.7% and a specificity of 75.0%, with the area under the curve (AUC) at 0.79 (95% CI: 0.72–0.86; *P*<0.001). With an AUC of 0.79, HOMA-IR showed a significantly greater discriminatory ability to predict poor outcome as compared with Hs-CRP (AUC: 0.64; 95% CI, 0.58–0.72; *P*<0.001), age (0.62; 0.55–0.69; *P*<0.001), and FBG (0.70; 0.64-0.76; *P*<0.01), while it was in the range of NIHSS score (AUC: 0.77; 95% CI: 0.70–0.75; *P*=0.56). In an ROC analysis of poor outcome, the AUC increased from 0.81 to 0.85 (95% CI: 0.80–0.89) by adding HOMA-IR to clinical examination variables (Table 4). A significant difference in the AUC between the clinical variables alone and the addition of HOMA-IR was observed (difference, 0.04 (95% CI: 0.02–0.06);* P*=0.02). The NRI statistic showed that the addition of HOMA-IR to established risk factors significantly increased the correct reclassification of poor outcome and good outcome patients (*P*=0.006). The IDI statistics found that the HOMA-IR level significantly increased discrimination between poor outcome patients and good outcome patients (*P*=0.04).

**Table 4:** HOMA-IR at admission prediction of poor functional outcomes with AUROC

Outcomes	AUROC				NRI (P)	IDI (P)
	HOMA-IR	Risk factors^1^	Risk factors with IR	Incremental area (P)^2^		
At admission	0.79	0.81	0.85	0.04 (0.02)	0.11 (0.006)	0.06 (0.04)

Abbreviations: AUROC, Area under the receiver operating characteristic.

1 Established risk factors include: age, sex, BMI, infarct volume, NIHSS score, time from onset to blood collection, stroke syndrome, stroke etiology, pre-stroke treatment, physical activity, vascular risk factors, and serum levels of Hs-CRP, FBG, HDL, LDL, and triglycerides.

2 Comparison of AUROCs: established risk factors without HOMA-IR compared with established risk factors with HOMA-IR.

## Discussion

In this prospective, population-based cohort study of nondiabetic individuals, we report that IR estimated using HOMA in Q4 compared with Q1–Q3 is associated with a 3.23-fold increased risk of poor functional outcome events. Adjustment for established cardiovascular risk factors, including glucose level, age, and NIHSS score, did not attenuate this association. Consistent with this finding, Jing et al. [19] showed that IR was associated with an increased risk of death, stroke recurrence, and poor outcome but not dependence in nondiabetic patients with acute ischemic stroke. In addition, Calleja et al. [20] confirmed that high IR may be associated with worse long-term outcome after acute ischemic stroke thrombolysis. Similarly, another study indicated that IR was an independent predictor of cardiovascular mortality in end-stage renal disease [21]. To our knowledge, this study is a novel finding and has not been previously described. It is imperative to emphasize targetted lifestyle intervention and more frequent medical interventions for stroke patients, especially for these patients with IR.

In a population-based cohort study amongst nondiabetic elderly, the authors reported that IR markers were not associated with risk of stroke or any of its subtypes [10]. Similarly, in this study, we also did not find an association between IR and stroke subtypes. In contrast, Urabe et al. [22] reported that impaired glucose tolerance and IR could play an important pathogenic role in the development of atherothrombotic infarction. Another study suggested that the IR score and central obesity were associated with incident lacunar disease [23]. The differences might have been caused by different settings of the studies, different follow-up times, different diagnostic methods, and different ethnicities studied.

IR may be a mechanism through which characteristics such as age, obesity, and inactivity lead to hypertension, stroke severity, and other factors, ultimately increasing stroke poor outcomes risk. However, IR still emerged as an independent predictor of a worse outcome after adjustment for those factors in logistic regression models. This suggests that IR may influence stroke outcomes through mechanisms other than those we considered in this article.

In this context, IR could play positive role in poor stroke outcomes by several potential mechanisms. First, patients with higher IR have elevated blood level of fibrinolysis inhibitors, such as plasminogen activator inhibitor 1, which may reflect an impairment of endogenous fibrinolytic capacity [24]. In a meta-analysis, a moderately strong association was found between usual plasma fibrinogen level and the risks of stroke, and nonvascular mortality in a wide range of circumstances in healthy middle-aged adults [25]. Second, IR may affect the structure of the offending clot itself, rendering the clot more dense and resistant to lysis. Moreover, the clots obtained from patients with metabolic syndrome are composed of thicker fibers and have more prolonged lysis times than the ones from individuals without increased IR [26]. Third, IR is associated with development of hypercoagulability [5]. A previous study had demonstrated that hypercoagulability was an important prognostic factor in stroke [27]. At last, IR may promote various conditions that could contribute to stroke outcome in general, such as increased platelet activation, endothelial dysfunction, and a chronic proinflammatory state, amongst others [4]. IR is frequently associated with endothelial dysfunction and has been proposed to play a major role in CVDs [28]. During insulin-resistant conditions, pathway-specific impairment in PI3K-dependent signaling may cause imbalance between production of nitric oxide (NO) and secretion of endothelin-1 and lead to endothelial dysfunction. A previous study suggested that fewer number of endothelial progenitor cells on admission is an independent risk factor for poor 6-month outcome in patients with ischemic stroke [29].

## Strengths and limitations

To avoid the confounding influence of glycemia, patients presenting with acute hyperglycemia were excluded from our study. Further, we chose a different strategy using the fourth quartiles, a more complete understanding of the effect of IR on the distribution of stroke outcomes can be obtained. At last, IDI and NR indices were calculated to determine the clinical utility of the addition of HOMA-IR to established risk factors and the ability of HOMA-IR to improve unfavorable outcome prediction.

The present study has some limitations. First, the relatively small sample size (*n*=173) may limit the generalization of the results of the present study. Before broad implementation, additional studies are needed for external validation. Second, we used the HOMA-IR to quantitate IR and not the gold standard hyperinsulinemic–euglycemic clamp because of practical limitations derived from our clinical setting. However, our selected method has been shown to correlate reasonably well with clamp-derived values [17]. Third, fasting blood samples used to determine HOMA-IR were obtained during the first 24 h after stroke onset and only once. At this timing, glucose metabolism may be inadequate with stroke insults. In addition, a determination of HOMA-IR at metabolic steady state (i.e. after resolution of any acute stress response) might be more informative about a direct role of IR in determining prognosis. Howver, HOMA-IR was measured only at one-time point (at admission). Serial measurements of HOMA-IR were not available because this was not considered during the planning of the cohort study. Were there any changes in HOMA-IR after admission, and were later values of HOMA-IR (such as 14 days after stroke onset) related to poor stroke outcomes need to be further assessed. Fourth, we only evaluated stroke outcomes at the subjects’ discharge. Previous studies [30,31] had confirmed that primary outcome as mRS at discharge was an appropriate time point for prognostic analysis of stroke. However, further study should be carried out to assess the association between HOMA-IR and stroke long-term outcomes. At last, the observational study does not allow advancing any cause and effect relationships.

## Conclusion

High HOMA-IR index is associated with a poor functional outcome in nondiabetic patients with acute ischemic stroke. IR may lead to a derangement of endogenous fibrinolysis, increased clot density, and lead to endothelial dysfunction, thus becoming a promising therapeutic target. It is imperative to emphasize targetted lifestyle intervention and more frequent medical interventions for stroke patients, especially for these patients with IR.

## Abbreviations

AUC: area under the curve
BMI: body mass index
CI: confidence interval
CVD: cardiovascular disease
FBG: fasting blood glucose
HOMA: homeostasis model assessment
Hs-CRP: high-sensitivity C-reactive protein
IDI: integrated discrimination improvement
IQR: interquartile range
IR: insulin resistance
mRS: modified Rankin scale
NIHSS: National Institutes of Health Stroke Scale
NRI: net reclassification improvement
OR: odds ratio
ROC: receiver operating characteristic curve
